# Rapid, automated, parallel quantitative immunoassays using highly integrated microfluidics and AlphaLISA

**DOI:** 10.1038/srep11339

**Published:** 2015-06-15

**Authors:** Zeta Tak For Yu, Huijiao Guan, Mei Ki Cheung, Walker M. McHugh, Timothy T. Cornell, Thomas P. Shanley, Katsuo Kurabayashi, Jianping Fu

**Affiliations:** 1 Department of Mechanical Engineering, University of Michigan, Ann Arbor, Michigan 48109, USA; 2 Department of Pediatrics and Communicable Diseases, University of Michigan, Ann Arbor, Michigan 48109, USA; 3 Department of Electrical Engineering and Computer Science, University of Michigan, Ann Arbor, Michigan 48109, USA; 4 Department of Biomedical Engineering, University of Michigan, Ann Arbor, Michigan 48109, USA

## Abstract

Immunoassays represent one of the most popular analytical methods for detection and quantification of biomolecules. However, conventional immunoassays such as ELISA and flow cytometry, even though providing high sensitivity and specificity and multiplexing capability, can be labor-intensive and prone to human error, making them unsuitable for standardized clinical diagnoses. Using a commercialized no-wash, homogeneous immunoassay technology (‘AlphaLISA’) in conjunction with integrated microfluidics, herein we developed a microfluidic immunoassay chip capable of rapid, automated, parallel immunoassays of microliter quantities of samples. Operation of the microfluidic immunoassay chip entailed rapid mixing and conjugation of AlphaLISA components with target analytes before quantitative imaging for analyte detections in up to eight samples simultaneously. Aspects such as fluid handling and operation, surface passivation, imaging uniformity, and detection sensitivity of the microfluidic immunoassay chip using AlphaLISA were investigated. The microfluidic immunoassay chip could detect one target analyte simultaneously for up to eight samples in 45 min with a limit of detection down to 10 pg mL^−1^. The microfluidic immunoassay chip was further utilized for functional immunophenotyping to examine cytokine secretion from human immune cells stimulated *ex vivo*. Together, the microfluidic immunoassay chip provides a promising high-throughput, high-content platform for rapid, automated, parallel quantitative immunosensing applications.

Immunoassays refer to a set of techniques that exploit the sensitivity and specificity of antibody-antigen interactions for detection of target analytes in biological samples^1^,^2^. Immunoassays play vital roles for detection and quantification of proteins and small biomolecules in medical diagnostics, proteomics, and pharmaceutical and biological research. Conventional immunoassays, which rely on enzyme-linked immunosorbent assay/spot (ELISA/ELISpot) and flow cytometry using encoded microbeads, offer high sensitivity and specificity. However, ELISA/ELISpot performed in microtiter plates are laborious, time-consuming, and suffer from intra- and inter-assay variations, making them unreliable as standardized assays in the clinical setting, particularly when time-critical analyte determination is warranted. Because they require multiple washes, ELISA/ELISpot are difficult to adapt to high-throughput operation and automation. Flow cytometry using encoded microbeads can achieve superior detection sensitivity, and the small laser detection volume allows no-wash assays to be conducted without the need to remove excess fluorescent tags on unbound probes. However, a flow cytometric instrument can be expensive, and requiring bulky, sophisticated free-space optics for multi-optical band detection, its whole functions cannot be readily incorporated into an integrated total analysis system. In addition, flow cytometry requires large sample volumes per assay, limiting serial (*e.g*. daily or more frequently) testing in pediatric and neonatal patients.

Recently, microfluidic immunoassays have gained significant attention owing to intrinsic advantages offered by microfluidic assay platforms^3^,^4^, which include: (1) increased surface-to-volume ratio advantageous for surface-bound antigen-antibody interactions; (2) reduced consumption of biological reagents and precious samples; (3) automated fluid handling and assay protocols that can improve assay reproducibility; (4) parallel designs for achieving high-throughput operation; and (5) integration with upstream sample preparation components on a single-chip platform for a completely integrated microscale total analysis system (μTAS) ideal for point-of-care (POC) tests. Furthermore, recent development of highly integrated microfluidics with multi-layered polymeric monoliths embedded with microvalves and micropumps significantly expands microscale fluid manipulation and assay performance to a greater capacity, precision, and complexity level^5^,^6^. Highly integrated microfluidics has been successfully applied in different chemical and biomedical applications, such as single-cell genomic and proteomic analysis^7^,^8^, stem cell culture^9^,^10^,^11^, and drug screening assays^12^,^13^. Nonetheless, till now, only a handful of integrated microfluidic platforms have been developed that can achieve automated, multiplex immunoassays entirely on-chip without any off-chip interventions^14^,^15^,^16^,^17^,^18^,^19^,^20^,^21^,^22^. Majority of these integrated microfluidic immunoassay chips rely on heterogeneous sandwich immunoassays for generating biosensing signals. While achieving a limit of detection (LOD) comparable to conventional ELISA, these integrated microfluidic immunoassay chips still require immobilizing capture antibodies on a solid phase and multiple washing, labeling, and signal amplification steps, retaining limitations associated with conventional ELISA in terms of assay complexity and time^14^,^15^,^16^,^17^,^18^,^19^,^20^,^21^,^22^.

Here, we developed a highly integrated microfluidic immunoassay chip incorporating a newly developed immunoassay technology, termed AlphaLISA (from PerkinElmer)^23^,^24^,^25^,^26^, to achieve rapid, automated, parallel immunoassays with microliter quantities of samples. As a no-wash, homogeneous bead-based sandwich immunoassay, AlphaLISA eliminates washing and blocking steps required for heterogeneous sandwich immunoassays such as ELISA that often result in analyte dilution and human errors. Immunosensing using AlphaLISA simply involves sequential conjugation of target analytes with anti-analyte acceptor beads and biotinylated antibodies and then streptavidin-coated donor beads in a free-solution environment (Fig. 1a). In the presence of target analytes, AlphaLISA donor and acceptor beads (according to the supplier, both are 250–350 nm in diameter) will thus be brought into close proximity (Fig. 1b). Excitation of AlphaLISA donor beads at 680 nm generates singlet oxygen molecules that trigger chemiluminescence emission at 615 nm from AlphaLISA acceptor beads (Fig. 1b). Chemiluminescence emission from AlphaLISA acceptor beads can then be measured to determine target analyte concentration. Using conventional microtiter plate assay platforms, the AlphaLISA technology requires only a small sample volume (down to 10 μL) while retaining the capability of achieving an excellent LOD down to sub-pg mL^−1^ and a large dynamic range of five orders of magnitude, comparable to immunosensing performance of state of the art ELISA methods.

To achieve rapid, automated, parallel immunoassays using integrated microfluidics in conjunction with the AlphaLISA technology, in this work a well designed and constructed microvalve infrastructure and its optimal operation for efficient fluid mixing and transport were achieved and incorporated into the microfluidic chip design. For reliable, repeated use of the microfluidic immunoassay chip, we further examined the effect of surface passivation on minimizing non-specific surface adsorption and background noise using the AlphaScreen Omnibead technology (from PerkinElmer), which as recommended by the manufacturer is ideal for characterizing instrument-related AlphaLISA assay variability (see Methods and Materials). Using the microfluidic immunoassay chip with the AlphaLISA technology, we demonstrated detection of one target analyte simultaneous from up to eight samples in 45 min with a LOD down to 10 pg mL^−1^. To demonstrate its potential clinical application, the microfluidic immunoassay chip was further utilized for functional immunophenotyping to examine cytokine secretion from human immune cells stimulated *ex vivo*.

## Methods And Materials

### Chip fabrication

The integrated microfluidic immunoassay chip was fabricated following protocols described previously^9^,^27^. Briefly, photolithography was used to pattern photoresist on two Si wafers as flow layer and control layer molds. Photolithography was conducted three times to form multilayered photoresist structures on the flow layer Si mold. The first and second photoresist layers were generated using SU8 (Microchem, Westborough, MA) and were 30 μm and 55 μm thick, respectively. The last photoresist layer was 20–30 μm thick (AZ50XT, Capitol Scientific, Austin, TX) and was rounded using reflow treatment at 130° C for 30 min. In parallel, the Si mold for control layer was fabricated to contain a stack of two SU8 layers, which were 35 μm and 60 μm thick, respectively. It should be noted that the second SU8 layer on both Si molds were spin-coated right after post-exposure bake of the first SU8 layer. Moreover, as the overall photoresist structures were very thick (~100 μm), there was a final long bake step to improve their evenness before using them as molds in soft lithography. Finally, the two Si molds were conformably coated with a 0.5 μm Parylene C dielectric layer at 690 °C and 30 mTorr (Specialty Coating Systems, Indianapolis, IN).

Soft lithography was carried out on the two Si molds as described previously^9^,^27^. Briefly, a 5 mm thick polydimethylsiloxane (PDMS; Dow Corning, Ellsworth, Germantown, WI) with a 10:1 monomer to curing agent ratio was poured over the flow layer Si mold, degassed, cured at 80 °C for 30 min, and peeled off the Si mold. In parallel, a 50 μm thick control PDMS layer with a 20:1 monomer to curing agent ratio was spin coated on the control layer Si mold before baked at 80 °C for 30 min. Dices cut from the flow PDMS layer were cleaned and treated with air plasma (Femto Science, Gyeonggi-Do, Korea) before bonded to the control PDMS layer under careful alignment. After baking at 80 °C for 30 min to complete irreversible bonding, the PDMS stack was peeled off from the control Si mold and hole-punched. The PDMS stack was treated with air plasma before bonded onto a glass slide pre-coated with PDMS of 10:1 monomer to curing agent ratio. The final microfluidic chip was baked at 80 °C overnight to ensure complete PDMS polymerization and strengthen bonding between different PDMS layers.

### Microfluidic control

Microvalves and micropumps integrated into the microfluidic immunoassay chip were operated using three modules: an external flow system, an electronic system, and control software. Compressed air was regulated to provide pressure to the overall flow control of the chip. 40 psi and 2–15 psi pressures were used to actuate microvalve closure and generate static pressure inside the chip, respectively. Pressure supply to actuate individual channels was regulated by an electronic system comprising a voltage generator (National Instruments, Austin, TX) with output signals connected to a set of electrically addressable pneumatic valve manifolds and regulators (Festo, Arlington Heights, IL and SMC, H.H. Barnum, Brighton, MI). LabVIEW (National Instruments) installed in a desktop computer was used to program the voltage generator.

### Immune cells and cell stimulation

A human monocytic cell line THP-1 (ATCC, Manassas, VA) derived from an acute monocytic leukemia patient was used for cytokine secretion assays. Cell culture medium comprised the base medium RPMI1640 (Life Technologies, Grand Island, NY), 10% fetal bovine serum (FBS; Fisher Scientific, Pittsburgh, PA), and 0.05 mM 2-mercaptoethanol. Floating THP-1 cells were maintained in T25 or T75 tissue culture flasks at 37 °C and 5% CO_2_ and were passaged every 3–4 days. Before cytokine stimulation, THP-1 cells were left intact overnight to minimize artifacts and assay variations.

We further obtained peripheral blood mononuclear cells (PBMCs) directly from healthy human blood for cytokine secretion assays. Institutional Review Board (IRB) of the University of Michigan, Ann Arbor approved the blood collection process and the methods were carried out in accordance with the approved guidelines. Informed consents were obtained from healthy donors before blood collection. Briefly, fresh blood was spiked with anticoagulant. After layering a density gradient medium Ficoll on the blood, the blood samples were centrifuged. The buffy coat containing PBMCs was carefully collected and assayed within 4 hr of blood collection.

Immune cells were stimulated off chip for secretion of immunoreactive cytokines such as tumor necrosis factor alpha (TNF-α) secretion using lipopolysaccharides (LPS; Sigma-Aldrich, Saint Louis, MO). LPS solution was first diluted in cell culture medium before added into vials containing cells. Vials were mounted onto a rotator (pluriPlix, pluriSelect Life Science, San Diego, CA) before the entire setup was incubated at 37 °C and 5% CO_2_ for 2 hr. Vials were centrifuged, and supernatants containing cell-secreted cytokines were transferred into another vial before loaded into the microfluidic immunoassay chip.

### ELISA

TNF-α concentrations were determined using a commercially available ELISA antibody pair kit (Life Technologies, Camarillo, CA). Briefly, Immulon 4HBX 96-well plates (Thermo Scientific, Waltham, MA) were coated with 2 μg mL^−1^ capture antibody in the DPBS buffer (137.0 mM NaCl, 8.0 mM Na_2_HPO_4_, 1.5 mM KH_2_PO_4_, 2.7 mM KCl, 0.1% ProClin™, pH = 7.4) over night at 4 °C. Plates were washed and then blocked with the DPBS buffer containing 0.1% (*v*/*v*) Tween-20 and 0.5% bovine serum albumin (BSA) fraction V (Fisher Scientific, Fair Lawn, NJ) for 1 hr. Microtiter plates were then loaded with recombinant human TNF-α samples spiked in the DPBS buffer and incubated at room temperature for 2 hr with 0.32 μg mL^−1^ detection antibody. Following incubation, microtiter plates were washed and then incubated with streptavidin-horseradish peroxidase (HRP) conjugates for 30 min at room temperature. Microtiter plates were washed and incubated with TMB stabilized chromagen solution (Life Technologies, Camarillo, CA) for 20 min, before the reaction was stopped using 1.8N H_2_SO_4_ solution. Absorbance of each well at a wavelength of 450 nm was measured using the M3 SpectraMax multi-mode plate reader (Molecular Devices, Sunnyvale, CA).

### Imaging and analysis

Imaging was conducted using a custom-built fluorescence microscope (Eclipse Ti-U, Nikon, Melville, NY) equipped with a 500 mW, 680 nm laser (Changchun New Industries, Changchun, China) and an electron-multiplying CCD (EMCCD) camera (Photometrics, Tucson, AZ). A free-space laser setup directed laser light through a 680 nm excitation filter (Semrock, Rochester, NY), a beam splitter, and a 2× objective to project into a uniform illumination of 4 mm × 4 mm. During imaging, the microfluidic immunoassay chip was placed on the microscope stage, with the imaging chamber array focused and aligned with the laser beam and the field of view (FOV) of the EMCCD camera. A mechanical shutter (Sutter, Novato, CA) was used to control laser pulse and exposure time. Fluorescent signals emitted from the imaging chamber array passed through a 615 nm emission filter (Semrock) before recorded by the EMCCD camera. In all experiments, laser excitation time was 0.5 sec, and EMCCD exposure time was 4.5 sec. Software NIS-Elements BR (Nikon) was used to program imaging parameters as well as to perform image analysis. To determine fluorescence intensity for each imaging chamber, eight regions of interest (ROI) overlaying the imaging chamber array were defined on fluorescent images to obtain average fluorescence intensity. Another eight ROIs adjacent to the imaging chamber array and known to contain no fluorescence source were defined in parallel to extract fluorescence background for each ROI. These backgrounds were then subtracted from fluorescence intensities of corresponding imaging chambers to yield final net fluorescence intensity for quantitative immunosensing.

### Microfluidic immunoassay

AlphaScreen Omnibead and AlphaLISA kits were both purchased from PerkinElmer (Waltham, MA). Given their light sensitivity, these reagents were stored and handled under filtered and subdued lighting. Rubrene-containing Omnibeads have all chemical components required for generation of chemiluminescence signal, and they are recommended by the supplier for characterizing instrument-related AlphaLISA assay variability. Thus, in this work, Omnibead solutions were used for characterizing imaging uniformity and reliability of the microfluidic immunoassay chip for repeated assays. Briefly, Omnibead solutions with known Omnibead concentrations (1.58–5 mg mL^−1^) were directly injected into the imaging chamber array of the microfluidic immunoassay chip. Such an Omnibead concentration range was chosen to generate a chemiluminescence signal comparable to that of AlphaLISA. After imaging, Omnibead solutions were evacuated using pressurized air before a blocking solution consisting of 10% BSA and 1% Tween-20 (both from Sigma-Aldrich) was injected to clean imaging chambers. The blocking solution was evacuated using pressurized air, and imaging chambers were dried off before imaged to record fluorescent signals. Fluorescent images were recorded for the imaging chamber array before and after three Omnibead solution filling and cleaning cycles.

For AlphaLISA cytokine measurements, the microfluidic immunoassay chip was first passivated with the blocking solution for 30 min at room temperature. The chip was then filled with AlphaLISA reagents (AlphaLISA donor beads, 5 mg mL^−1^; AlphaLISA acceptor beads, 1.2 mg mL^−1^; biotinylated antibodies, 125 nM) and sample solutions containing analytes to be assayed. Microfluidic immunoassays were then conducted using the AlphaLISA components. After imaging, the blocking solution was used to clean imaging chambers. Since AlphaLISA donor beads are light sensitive, the chip pre-filling step and subsequent immunoassay operations were conducted in a subdued and green-filtered lighting condition.

## Results And Discussion

### Design and operation of the microfluidic immunoassay chip

The microfluidic immunoassay chip was integrated with microvalves and micropumps to achieve rapid fluid transport and mixing and conjugation of target cytokines with AlphaLISA assay components. Patterned from the two microfabricated Si molds, the microfluidic immunoassay chip comprised two PDMS layers, with the top layer for transporting fluid samples and reagents (“flow layer”) and the bottom layer embedded with a thin membrane and pneumatic pads for controlling fluid transport in the top flow layer (“control layer”). Fluid channels with round cross-sections were fabricated in the top flow layer, allowing complete closure of fluid channels by actuating pneumatic pads as isolation valves in the bottom control layer. A series of three pneumatic pads in the bottom control layer were actuated with a proper rhythm for active metering and pumping flow in the top flow layer^27^.

**Figure 1:**
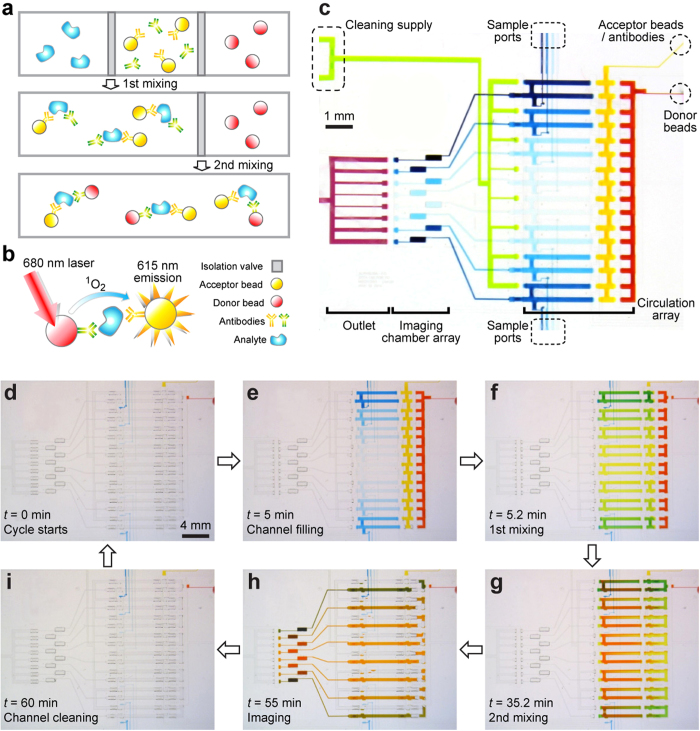
Integrated microfluidic immunosensing chip for rapid, automated, and parallel cytokine detection from biological solutions. (**a**) Schematic of AlphaLISA bead conjugation. Initially, analytes and AlphaLISA assay components were separated through isolation valves. Programmed micropumps promoted the first mixing step between analytes, anti-analyte AlphaLISA acceptor beads, and biotinylated antibodies, before the second mixing with streptavidin-coated AlphaLISA donor beads. (**b**) Principle of AlphaLISA immunosensing. (**c**) Chip layout. The two-layered PDMS microfluidic chip was integrated with microvalves and micropumps for flow isolation, metering, and pumping. The chip consisted of two main regions: a circulation array for homogeneous mixing and conjugation of cytokines (injected from eight sample ports in *blue*) with AlphaLISA components (streptavidin-coated AlphaLISA donor beads in *red* and anti-analyte AlphaLISA acceptor beads and biotinylated antibodies in *yellow*) and an imaging chamber array that centralized and amplified AlphaLISA fluorescent signals. Both the circulation and imaging chamber arrays comprised eight identical replicates of circulation loop and imaging chamber, respectively, allowing for eight different samples (as illustrated by different blue colors) processed and detected simultaneously. Furthermore, a programmable cleaning scheme which flushed through chip compartments with air and blocking solution (in *green*) *via* a common outlet (in *purple*) was integrated into the chip to help recover the chip for repeated AlphaLISA immunoassays. (**d**–**i**) Chip operation. Flow visualization and operation optimization were conducted using food dyes. Initially, compartments of the microfluidic chip were all empty (**d**). The circulation array was first filled with analyte solutions (in *blue*), AlphaLISA acceptor beads / antibodies (in *yellow*), and AlphaLISA donor beads (in *red*) (**e**). The first mixing step was then carried out to mix analytes and AlphaLISA acceptor beads / antibodies (**f**). After 20 sec of the first mixing process, solution color appeared very even, suggesting that mixing could be achieved rapidly. The second mixing was performed to mix analytes, AlphaLISA acceptor beads / antibodies, and AlphaLISA donor beads (**g**). AlphaLISA conjugation mixtures were then transported to the imaging chamber array for detection (**h**). Finally, a cleaning process was carried out to remove all solutions and recover the chip to the initial clean state before next cycles were initiated (**i**).

The top flow layer of the microfluidic immunoassay chip contained two main regions: a circulation loop array for rapid, homogeneous mixing and conjugation of target analytes in sample solutions with AlphaLISA assay components and an imaging chamber array that centralized and amplified fluorescent signals from AlphaLISA assays (Fig. 1c). Both the circulation loop and imaging chamber arrays comprised eight identical replicates, allowing for eight different samples processed and assayed simultaneously. Using microvalves, each circulation loop was divided into three compartments that were filled with (1) sample solutions containing target analytes, (2) anti-analyte AlphaLISA acceptor beads (1.2 mg mL^−1^) and biotinylated antibodies (125 nM), and (3) streptavidin-coated AlphaLISA donor beads (5 mg mL^−1^) (Fig. 1c). Microvalves were regulated to allow active mixing between analyte solutions and AlphaLISA assay components (Fig. 1a). Since light intensity detected at 615 nm was proportional to analyte concentration, we could measure fluorescence intensity of AlphaLISA assay products using a custom-built laser detection setup to quantitatively measure concentration levels of target analytes (Fig. 1b)^28^,^29^. With via structures to connect fluid channels between the flow and control layers^30^, the eight circulation loops arranged in parallel could share reagents such as AlphaLISA reagents and cleaning solution. The imaging chambers were all confined within a 4 mm × 4 mm field of view (FOV) to be examined simultaneously under the laser detection system. It should be noted that the height of imaging chambers was significantly greater than fluid channels in the top flow layer, in order to enhance fluorescent signals and thus the LOD of AlphaLISA assays on chip.

We first characterized and optimized AlphaLISA assay operation in the microfluidic immunoassay chip by adding food dyes into AlphaLISA reagents and sample solutions for flow visualization (Fig. 1d–i). The chip was first pre-filled with AlphaLISA reagents and sample solutions up to microvalves, while both the circulation loop and imaging chamber arrays were empty (Fig. 1d). Once an immunoassay was initiated, eight sample solutions and AlphaLISA reagents were pushed into the circulation loops by a static pressure till corresponding compartments in the circulation loops were completely filled in 5 min (Fig. 1e). Air was completely evacuated during this step to ensure bubble-free operation. By actuating microvalves, the first circulation operation was conducted for 30 min to mix sample solutions and AlphaLISA acceptor beads / antibodies, while AlphaLISA donor bead solutions remained isolated (Fig. 1a,f). An optimized pumping rhythm was used for rapid fluid circulation to achieve homogeneous mixing in as short as 20 sec (Fig. 1f). After 30 min, the second circulation was initiated and conducted for 15 min to mix mixtures resulted from the first circulation and solutions containing AlphaLISA donor beads (Fig. 1g). In both circulation operations, the circulation speed was maximized when microvalves toggled in a 0.03 sec step. At this point the circulation loop array had completed its function, and final AlphaLISA conjugation mixtures were transported to the imaging chamber array, excited by a 0.5 W 680 nm laser and imaged for 4.5 sec for signal detection (Fig. 1h). Colors of the imaging chambers shown in Fig. 1h were significantly darker compared to colors of flow channels in the circulation loop array, illustrating a greater chamber height necessary to accumulate AlphaLISA conjugation mixtures to enhance imaging signals. Due to non-specific photobleaching, AlphaLISA conjugation mixtures contained in the eight imaging chambers were imaged simultaneously only once. To repeat immunoassays using the same microfluidic immunoassay chip, all solutions in the chip were first evacuated under pressurized air before the blocking solution containing 10% BSA and 1% Tween-20 was injected to clean the circulation loop and imaging chamber arrays (Fig. 1i). The cleaning solution was evacuated using pressurized air, and the imaging chambers were dried off before next operation cycles. The whole cleaning process took less than 5 min.

### Surface passivation and imaging uniformity

Surface passivation is important and necessary for quantitative immunosensing in a confined microfluidic environment, given large surface-to-volume ratio of a microfluidic environment and non-specific surface adsorption of biomolecules. In addition, it is known that even after long-term thermal curing procedure, untreated bare PDMS surfaces still contain dangling molecular structures as well as incompletely cured monomers, prone to increasing background noise for biosensing^31^. To prevent non-specific adsorption of biomolecules, which is especially detrimental for repeated immunoassays using the same device, the microfluidic immunosensing chip was first passivated with the blocking solution consisting 10% BSA and 1% Tween-20 for 30 min at room temperature.

**Figure 2:**
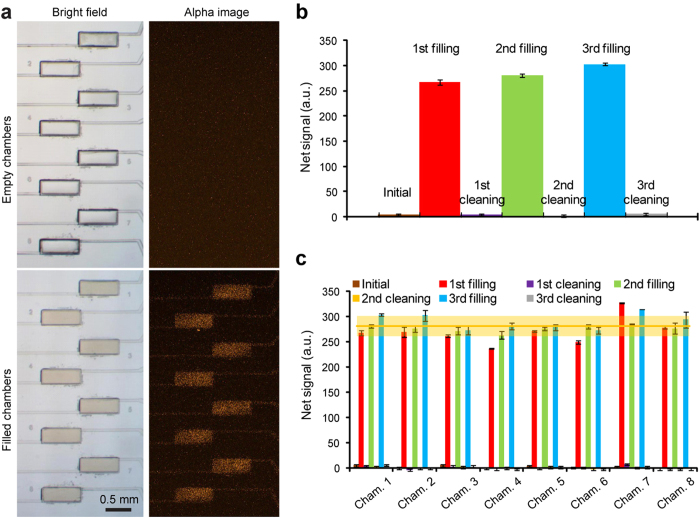
Reliability of the microfluidic immunosensing chip for repeated immunoassays. Surface passivation was achieved using BSA and Tween-20 to block compartments of the microfluidic immunosensing chip. (**a**) Brightfield (*left*) and fluorescent (*right*) images showing the imaging chamber array before (*top*) and after (*bottom*) being filled with Omnibead solutions (5 mg mL^**−**1^). (**b**) Representative plot of fluorescent signal detected from one imaging chamber before and after three Omnibead solution filling and cleaning cycles. (**c**) Bar plot of fluorescent signals collected simultaneously from all eight chambers of one imaging chamber array, where three Omnibead solution fillings followed by three chamber cleanings were carried out. The error bar for each chamber represents the range, and *n* = 2. The mean fluorescent signal (*yellow line*) ± one standard deviation (S.D.; *orange area*) from all Omnibead solution filled chambers were calculated and included in the plot.

To assess the effectiveness of surface passivation on non-specific surface adsorption, the microfluidic immunoassay chip was repeatedly filled with 5 mg mL^−1^ Omnibead solution before its subsequent evacuation and chip cleaning with the blocking solution. Fluorescent images of the imaging chamber array with or without Omnibead solutions were recorded and analyzed (Fig. 2a). Our data showed that after each evacuation and cleaning, net fluorescent signal detected for each imaging chamber was minimal and comparable to fluorescence background. Furthermore, there was no detectable increase of fluorescent signal for imaging chambers after each sequential filling and evacuation of 5 mg mL^−1^ Omnibead solutions (Fig. 2b,c), supporting that surface passivation with 10% BSA and 1% Tween-20 was efficient for preventing non-specific surface adsorption and retrieving the microfluidic immunoassay chip for repeated immunoassays.

**Figure 3:**
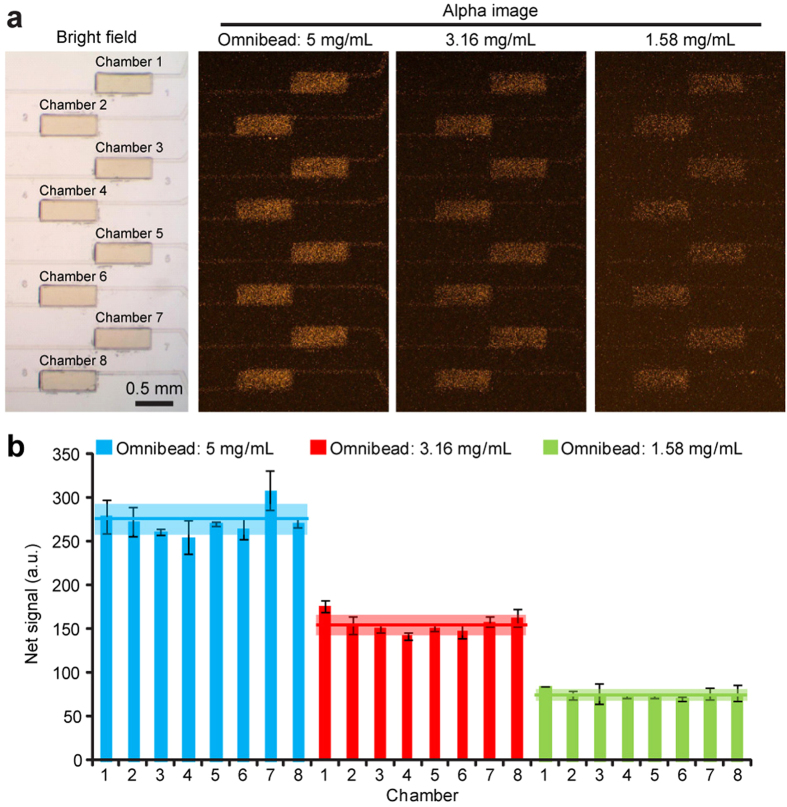
Biosensing uniformity of the microfluidic immunosensing chip. (**a**) Brightfield (*leftmost*) and fluorescent (*right*) images showing the imaging chamber array filled with Omnibead solutions of three different concentrations, as indicated. (**b**) Bar plot of fluorescent signals detected simultaneously from all eight chambers of one imaging chamber array filled with Omnibead solutions of three different concentrations, as indicated. Data for each chamber represents the mean ± S.D., and *n* = 3. The mean fluorescent signal (*colored line*) ± S.D. (*colored area*) calculated for the whole chamber array were included in the plot.

The eight identical imaging chambers were designed for high-throughput, parallel quantitative immunosensing. However, imaging variation could possibly result from non-uniform chamber geometries (due to chip fabrication process) as well as non-uniform laser illumination or EMCCD detection. We next performed assays to assess imaging uniformity across the imaging chamber array using Omnibead solutions of different concentrations (5 mg mL^−1^, 3.16 mg mL^−1^, and 1.58 mg mL^−1^; Fig. 3a). For 5 mg mL^−1^ Omnibead solution, the overall coefficient of variation (CV, defined as the ratio of standard deviation to mean) of fluorescence intensity detected for the imaging chamber array was about 7% (Fig. 3b). Similarly, under the two lower Omnibead concentration conditions, the overall CVs of fluorescent signals detected for the imaging chamber array remained as low as 7.5% and 8.6%, for 3.16 mg mL^−1^ and 1.58 mg mL^−1^ Omnibead concentrations, respectively (Fig. 3b). Together, our data supported excellent imaging uniformity of the microfluidic immunoassay chip, critical for parallel quantitative immunosensing. Our data also suggested that the microfluidic immunoassay chip should reliably differentiate samples containing target analytes with a concentration difference as small as 10%.

### Functional phenotyping of immune cells

Immune cell functional response is most often characterized by secretion of cytokines, which are cell-secreted proteins essential for intercellular signaling and regulation of immune cell maturation, growth, and responsiveness. In biomedical research and clinical settings, characterizing cytokine secretion patterns of human immune cells provides a powerful means to monitor human immunity that is critical for fundamental understanding of human immunology and developing therapeutic treatments for conditions such as allergy, asthma, autoimmunity, acquired and primary immunodeficiency, transplantation, and infection^32^,^33^.

**Figure 4:**
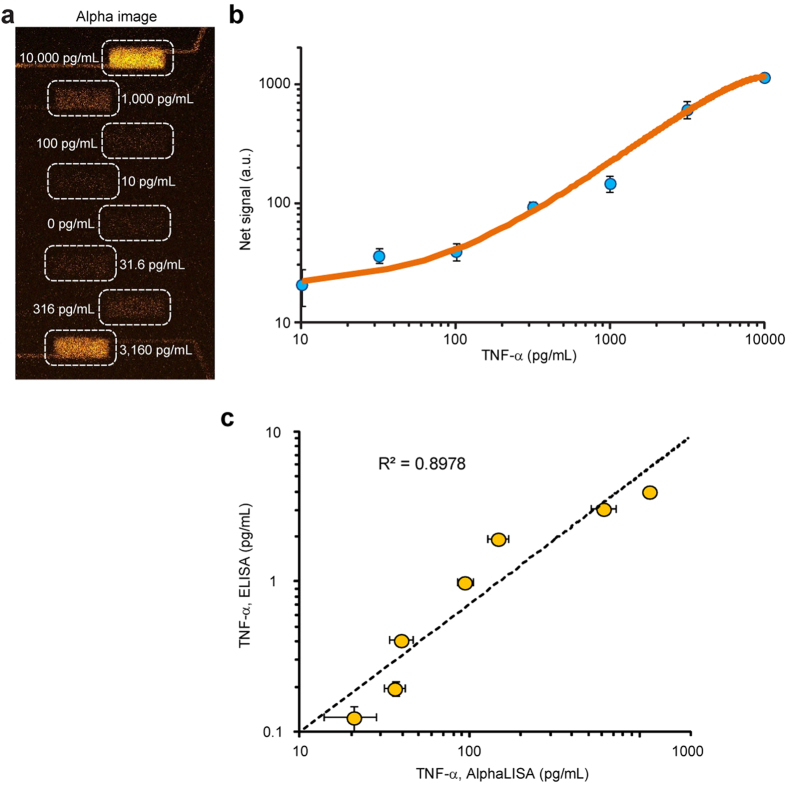
Standard calibration curve for TNF-α detection using the integrated microfluidic immunosensing chip. To generate the standard curve, cell culture medium spiked with TNF-α were titrated to obtain eight different concentrations spanning a broad range from 0–10,000 pg mL^**−**1^. All eight samples were assayed simultaneously using the microfluidic immunosensing chip to obtain their corresponding fluorescent signals. (**a**) Representative fluorescent image showing the imaging chamber array filled with cell culture medium containing TNF-α of eight different concentrations, as indicated. Chamber areas were highlighted with white dashed rectangles to guide the eye. (**b**) Fluorescent signal as a function of TNF-α concentration. Note that data from 0 pg mL^**−**1^ TNF-α solution was not plotted. (**c**) Correlation between AlphaLISA performed in the microfluidic immunosensing chip and ELISA. Data represents the mean ± S.D. (*n* = 3 for AlphaLISA in the chip and *n* = 2 for ELISA).

For quantitative immunosensing of cytokines, the integrated microfluidic immunoassay chip was first calibrated using control samples spiked with known concentrations of tumor-necrosis factor-α (TNF-α) covering a broad range from 0–10,000 pg mL^−1^ (Fig. 4a). TNF-α is a pro-inflammatory cytokine and key biomarker associated with host defense and immunosurveillance. TNF-α secretion from immune cells stimulated *ex vivo* with LPS has been shown to reflect function status of innate immunity. Control samples spiked with TNF-α and AlphaLISA reagents (AlphaLISA donor beads, 5 mg mL^−1^; AlphaLISA acceptor beads, 1.2 mg mL^−1^; biotinylated antibodies, 125 nM) were processed by the integrated microfluidic immunoassay chip using the optimal protocol descried in the previous section (Fig. 4a). Fluorescence intensity of each imaging chamber was then determined and plotted as a function of TNF-α concentration to establish a calibration curve (Fig. 4b). Our results in Fig. 4b further suggested that for the integrated microfluidic immunoassay chip, a LOD for TNF-α, defined as 3 times the standard deviation of the blank (with 0 pg mL^−1^ TNF-α), was about 10 pg mL^−1^, comparable to ELISA. Dynamic range for TNF-α measurement using the integrated microfluidic immunoassay chip spanned four orders of magnitude from 10 pg mL^−1^ to 10,000 pg mL^−1^ (Fig. 4b). Notably, our data in Fig. 4b showed CVs of fluorescence signals between 5%–16% for control samples spiked with TNF-α > 32 pg mL^−1^, suggesting that automated operation of the microfluidic immunoassay chip, in conjunction with integrated micropumps, allowed for active homogeneous mixing of analyte samples and AlphaLISA reagents and significantly improved assay reproducibility as compared to conventional immunoassays involving human interventions and static microplates or microvials. Since increasing AlphaLISA reagent concentration can increase immunosensing sensitivity, the AlphaLISA reagent concentrations used in this work were greater than recommended values by the supplier. Readers interested in AlphaLISA assay optimization can refer to studies published previously^34^,^35^,^36^,^37^.

To further evaluate the utility of the microfluidic immunoassay chip, TNF-α samples of the same concentrations spiked in PBS were titrated by ELISA, with results compared to the data measured using the microfluidic immunoassay chip. This comparison revealed a good correlation (*R*^2^ = 0.90) between the data obtained from the microfluidic immunosensing chip using AlphaLISA and the ELISA (Fig. 4c).

**Figure 5:**
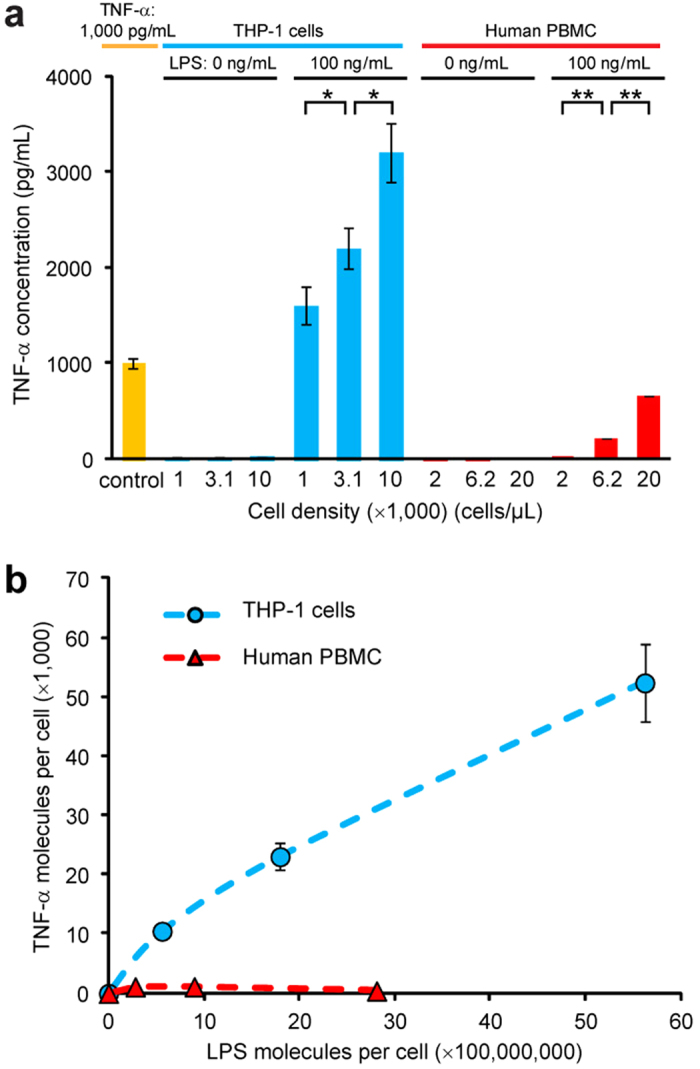
Detection of TNF-α secreted from THP-1 cells and human peripheral blood mononuclear cells (PBMCs) using the integrated microfluidic immunosensing chip. (**a**) Plot of TNF-α concentration as a function of THP-1 cell and human PBMC density. Immune cells were either untreated (LPS: 0 ng mL^**−**1^) or pre-stimulated with LPS (100 ng mL^**−**1^), as indicated. A cell culture medium spiked with 1,000 pg mL^**−**1^ TNF-α was included as positive control for comparison. (**b**) Plot of average TNF-α molecules secreted by individual THP-1 cells and human PBMCs as a function of LPS concentration per cell. Data represents the mean ± S.D., and *n* = 3. *P*-values were calculated using unpaired student’s *t*-tests. *,*P* < 0.05 and **,*P* < 0.005.

To assess utility of the integrated microfluidic immunoassay chip for quantitative immunophenotyping of immune cells, different densities of THP-1 cells (1,000, 3,100, and 10,000 cells μL^**−1**^) and human PBMCs (2,000, 6,200, and 20,000 cells μL^**−1**^) were stimulated with 100 ng mL^**−1**^ LPS off-chip for 2 hr, before supernatants containing cytokines were collected and assayed. For comparison, supernatants from the same densities of untreated THP-1 cells and PBMCs were also collected and assayed. To minimize experimental uncertainty, another positive control spiked with 1,000 pg mL^−1^ TNF-α was assayed and included as a standard for data normalization. Fluorescence signals were measured from the imaging chamber array and converted into effective TNF-α concentration using the calibration curve established in Fig. 4b. Figure 5a showed TNF-α concentration as a function of THP-1 cell and PBMC density and LPS treatment dose. As expected, level of TNF-α secreted by untreated THP-1 cells and PBMCs was below the LOD, even for a high cell density above 10,000 cells μL^**−1**^. THP-1 cells and PBMCs stimulated with 100 ng mL^**−**1^ LPS secreted detectable levels of TNF-α, even for cell densities as low as 1,000 and 2,000 cells μL^**−**1^, respectively. Though TNF-α secretion from PBMCs was comparatively low, TNF-α secretion increased with immune cell density under LPS stimulation. To further understand the effect of LPS stimulation on TNF-α secretion from immune cells, the average number of TNF-α molecules produced by single THP-1 cells and single human PBMCs was plotted as a function of the average number of LPS molecules available for each single immune cell (Fig. 5b). TNF-α secretion from single PBMCs was stable over different concentrations of LPS in the cell environment. In contrast, TNF-α secretion from THP-1 cells was highly proportional to the concentration of LPS in the cell environment.

The total immune cell density in blood from healthy humans is in the range of 4,000–7,000 cells μL^**−**1^, with some important immune cell subpopulations (*e.g*. T-cells and monocytes) having cell densities between 200–350 cell μL^**−**1^. A linear extrapolation of data in Fig. 5a for LPS-stimulated THP-1 cells to the 10 pg mL^**−**1^ LOD suggested that an immune cell density as low as 200 cells μL^**−**1^ was still feasible for TNF-α detection using the integrated microfluidic immunoassay chip.

## Conclusion

Conventional immunoassays including ELISA and flow cytometry are time-consuming, labor-intensive, and error-prone. To address these limitations, different microfluidic immunoassay platforms have been developed in the past with different levels of success for quantitative measurements of protein biomarkers in biological samples showing good correlations and comparable sensitivities to conventional ELISA^14^,^15^,^16^,^17^,^18^,^19^,^20^,^21^,^22^. However, majority of these microfluidic immunoassay chips still utilize heterogeneous sandwich immunoassays for biodetection, necessitating immobilized capture antibodies on a solid phase and multiple washing, labeling, and signal amplification steps that can increase assay complexity and time and result variation. In this work, we leveraged the no-wash, homogeneous bead-based AlphaLISA technology to develop a highly integrated microfluidic immunoassay chip capable of rapid, automated, parallel immunoassays. AlphaLISA eliminates washing and blocking steps required for heterogeneous sandwich immunoassays, and its operation in a free-solution environment also makes it ideal as an immunosensing mechanism in a microfluidic environment that can be conveniently manipulated using integrated microfluidic technologies.

The microfluidic immunoassay chip using AlphaLISA could detect one target analyte simultaneously from up to eight samples in 45 min with a LOD down to 10 pg mL^**−**1^ that is comparable to ELISA. The significantly shortened assay time as compared to conventional immunoassays, which typically require at least a few hours, was advantageous and desirable when considering point-of-care (POC) testing needs, real-time clinical decision making, and timely result determination for biomarker-based study stratification or randomization for *enriched* clinical trial design. Parallel biosensing capability and automated operation of the microfluidic immunoassay chip improved assay throughput, reduced reagent cost, and enhanced assay reproducibility for measurements of target analytes from a panel of biological samples. For example, for the entire assays to generate the TNF-α calibration curve (Fig. 4), only 4 μL TNF-α samples and AlphaLISA reagents were used with multiple assay cycles (>10).

Importantly, we demonstrated that surface passivation with 10% BSA and 1% Tween-20 was efficient for preventing non-specific surface adsorption and retrieving the microfluidic immunoassay chip for repeated immunoassays. This feature was achieved with the help of an integrated cleaning scheme that effectively removed residual samples from the chip before subsequent immunoassay cycles. Thus, each single microfluidic immunoassay chip, in principle, could be utilized for repeated assays over time. This feature is important for applications where real-time, dynamic information of biomolecule concentration variation is desired (such as dynamic studies of cytokine production from immune cells).

The microfluidic immunoassay chip was further utilized for functional immunophenotyping to examine cytokine secretion from human immune cells stimulated *ex vivo*. It should be noted that assessment of patient immune status based on measurements of cytokine production of immune cells is among the most commonly employed and clinically proven methods for assessing patients’ immune status. Our microfluidic immunoassay platform leveraging highly integrated microfluidics for assay automation allowed rapid, accurate, and parallel immunoassays on small amounts of immune cells (down to 200 cells μL^**−**1^), providing a promising platform for systems-level studies of human immunity and clinical diagnosis of immune disorders and diseases. Future optimization of immunosensing protocols and integration of upstream blood cell sorting components into the microfluidic immunoassay chip should further improve immunoassay sensitivity of virtually any specific cell population, enhance throughput, and shorten assay time^38^,^39^. Together, we believe that the microfluidic immunoassay chip demonstrated in this work provides a promising high-throughput, high-content platform for rapid, automated, parallel quantitative immunosensing applications.

## Additional Information

**How to cite this article**: Zeta Tak For Yu *et al*. Rapid, automated, parallel quantitative immunoassays using highly integrated microfluidics and AlphaLISA. *Sci. Rep*. **5**, 11339; doi: 10.1038/srep11339 (2015).
